# Intention Attribution in Children and Adolescents with Autism Spectrum Disorder: An EEG Study

**DOI:** 10.1007/s10803-021-05358-1

**Published:** 2021-12-02

**Authors:** Magdalena Schütz, Sara Boxhoorn, Andreas M. Mühlherr, Hannah Mössinger, Christine M. Freitag, Christina Luckhardt

**Affiliations:** Department of Child and Adolescent Psychiatry, Psychotherapy and Psychosomatics, University Hospital of the Goethe-University, Frankfurt, Germany

**Keywords:** Autism, EEG, Social cognition, Intention attribution, Expectation violation, Predictive coding

## Abstract

The ability to infer intentions from observed behavior and predict actions based on this inference, known as intention attribution (IA), has been hypothesized to be impaired in individuals with autism spectrum disorder (ASD). The underlying neural processes, however, have not been conclusively determined. The aim of this study was to examine the neural signature of IA in children and adolescents with ASD, and to elucidate potential links to contextual updating processes using electroencephalography. Results did not indicate that IA or early contextual updating was impaired in ASD. However, there was evidence of aberrant processing of expectation violations in ASD, particularly if the expectation was based on IA. Results are discussed within the context of impaired predictive coding in ASD.

**Supplementary Information:**

The online version contains supplementary material available at 10.1007/s10803-021-05358-1.

## Introduction

Autism spectrum disorders (ASD) are characterized by impaired social communication as well as stereotyped and repetitive behaviors and interests (American Psychiatric Association, 2013). A recent review on cognitive impairments associated with ASD found that *mentalizing abilities* were consistently attenuated in adults with ASD (Velikonja et al., 2019). *Mentalizing* summarizes a set of skills including abilities such as intention- or mental-state attribution, which are crucial for successful social interaction. Intention-attribution (IA), which falls under *mentalizing abilities,* for instance, helps us understand and interpret other people’s behavior, or predict their next action. Impairments in *mentalizing abilities*—particularly in IA—have been hypothesized to underlie deficits in social interactions and communication in indivduals with ASD (e.g. Lombardo et al., 2011; Mason et al., 2008). Studies examining IA in participants with ASD have often reported impairments in this ability (e.g. Schneider et al., 2013; Schuwerk et al., 2016; Vivanti et al., 2011; Williams & Happé, 2010), but findings are inconsistent (e.g. Ciaramidaro et al., 2015). In addition, the neural processes underlying IA remain understudied, particularly in ASD, although there is evidence that activation in brain areas associated with mentalizing abilities differs between participants with and without ASD (e.g. Ciaramidaro et al., 2015; Kana et al., 2014; Murdaugh et al., 2014). Examining the underlying neural mechanisms could, therefore, provide important insights into how individuals with ASD process IA and whether this could impact their mentalizing ability.

A well-established method for investigating IA in the laboratory setting is the sequential comic strip paradigm (e.g. Vistoli et al., 2011, 2015). Participants view comics that represent the different stages of an action or event and are asked to identify the congruous ending. The control condition, physical causality (PC) is very similar to IA, as it involves a sequence of events that logically lead to a certain outcome, but at the same time do not involve a person’s intentional actions. Predictions in the IA condition are based on the intention that is attributed to the character in the comic; predictions in the PC condition are based on various physical properties of objects and laws of physics, such as gravity. Therefore, both conditions draw on the same cognitive processes related to the processing of complex sequences of events and anticipation of consequences, but at the same time allow for the isolation of the processes that uniquely underlie IA.

Studies examining this paradigm using positron emission tomography (PET; Brunet et al., 2000) and functional magnetic resonance imaging (fMRI; Ciaramidaro et al., 2007; Walter et al., 2004) in typically developing (TD) young adults have found differential activation patterns for IA compared to PC conditions with significantly increased activation for IA in frontal and temporal areas relevant for IA, such as the medial prefrontal cortex (mPFC) and temporo-parietal junction (TPJ).

In adults with ASD, fMRI studies comparing IA and PC with the sequential comic strip paradigm have found reduced activation compared to TD participants during IA in the TPJ, rIFG and left premotor cortex (PMC; Ciaramidaro et al., 2015; Kana et al., 2014; Murdaugh et al., 2014), accompanied (with the exception of Ciaramidaro et al., 2015) by an increased error rate for IA, but not PC trials. These findings indicate that attenuated IA performance in participants with ASD is associated with decreased activation in key brain regions underlying IA processing. However, due to the poor temporal resolution of fMRI, it remains unknown at which steps of IA processing these differences occur and which exact neural processes underlie IA impairments in individuals with ASD.

Neurophysiological methods, such as electroencephalography (EEG) or magnetoencephalography (MEG) can be used to determine a more precise time course of the neural signal underlying IA. Studies using MEG (Vistoli et al., 2011) and EEG (Vistoli et al., 2015) have implemented the comic strip paradigm in TD adults. In these studies, the comics were presented sequentially in order to compare neural activation between images, i.e. different processing stages related to IA. These comics were designed so that during the presentation of the 3rd image, the outcome of the story depicted in the comic could be predicted, either based on IA (i.e. the goal of the actor became clear) or PC (i.e. it became evident what would happen to the depicted object). Looking specifically at the 3rd image, they found that IA was associated with a significantly stronger positive activation approximately 200–600 ms after picture onset in the right posterior superior temporal sulcus (pSTS) and right TPJ, as well as the right inferior parietal lobule (IPL) compared to the PC condition. Additionally, the right intraparietal sulcus (IPS) also showed increased activation 240–540 ms after stimulus onset for IA (Vistoli et al., 2011). Building on these findings, an EEG study found a significant difference between IA and PC in a positive component between 250–600 ms (with a peak at 300 ms) after onset of the 3rd image (i.e. enhanced amplitude for IA compared to PC) in bilateral posterior electrodes associated with IPL, TPJ and pSTS in TD adults (Vistoli et al., 2015). This effect was only found for the 3rd, but not the first or second image, suggesting that this component may reflect increased difficulty associated with contextual integration (i.e. integrating the information from previous images) required for processing IA compared to PC (Vistoli et al., 2015). Interestingly, the authors noted the similarity of this component with the P3-like component associated with context- and working-memory-updating processes (Friedman et al., 2001; Ibanez et al., 2012), and propose that IA is based on contextual integration (Brunet-Gouet et al., 2011). Implementing this paradigm in ASD, therefore, will provide a more detailed understanding of which processes are affected in ASD, and whether impaired IA is rooted in aberrant contextual integration processes. Aberrant contextual integration has been hypothesized to underlie other ASD symptoms, such as repetitive behaviors and sensory abnormalities, within the framework of predictive coding theory (PCT; e.g. Pellicano & Burr, 2012).

Furthermore, the comic strip paradigm can also be used to examine neural correlates of the processing of information that is either congruous or incongruous to one’s own expectations, which is not only another important aspect of PCT, but also known to be affected in ASD (van de Cruys et al., 2014). While previous ERP studies using the comic strip paradigm have not attempted to analyze neural processes related to the ending of the story, ERP studies applying other paradigms have demonstrated that several components related to incongruous information can be analyzed, as incongruous endings are expectation violations. Specifically, one ERP study examined ERPs for congruous vs. incongruous endings of stories and observed two distinct components (van der Cruyssen et al., 2009): a positive frontal component around 200 ms after stimulus onset at electrodes associated with the mPFC, that was larger for incongruous endings, likely reflecting a redirection of attention toward the unexpected information, and a positive component in parietal electrodes associated with the TPJ around 300 ms after stimulus onset that was stronger for incongruous endings. This latter component is also consistent with ERP research assessing congruous/incongruous endings based on trait inferences (e.g. Bartholomow et al., 2001; van Duynslaeger et al., 2007, 2008). It is supposed to reflect a deliberate integration of the unexpected information into the preceding contextual information. In addition, continuous differential neural activation between congruous and incongruous endings was observed in medial or lateral frontal electrodes between 600 and up to 1200 ms after stimulus onset. Given that individuals with ASD have difficulties processing expectation violations, such as incongruous endings (for a review, see van de Cruys et al., 2014), we aim to also examine activation during the 4th picture, in which the (congruous or incongruous) ending is shown.

In summary, the aim of the present study is to examine the P3-like ERP signature associated with IA during the presentation of the 3rd and 4th image of the sequential comic strip paradigm, and the later ERP associated with the processing of expectation violations during the presentation of the 4th image in participants with ASD. By comparing amplitude and latency of the ERPs we add time-related information on the neural processes underlying IA. In addition, we examine whether aberrant neural processing in ASD is specific to IA or may reflect a more general neural information processing deficit.

It should be noted that the abovementioned ERP-studies on IA and expectation violations have only examined adults. Maturation in areas relevant for social cognition, such as the mPFC, pSTS and TPJ, still takes place during adolescence (Blakemore, 2008, 2012; Choudhury et al., 2008; Dumontheil, 2015; Mills et al., 2014). This corresponds to reports of continuous improvement of social cognitive skills, for instance perspective-taking, face-processing ability and social attribution from childhood to early adulthood (Choudhury et al., 2006, 2008; Hu et al., 2010). In addition, an fMRI study contrasting activation between IA and PC in adolescents between 12 and 18 years and adults found a stronger activation in the mPFC in adolescence, while the difference in activation between IA and PC in adults was stronger in the right STS (Blakemore et al., 2007), indicating a flexible involvement of social brain areas during development. To test whether the effects found by Vistoli et al., (2011; 2014) could be replicated in children and adolescents, a pilot study with N = 6 children was conducted (see ‘Methods’).

Based on the findings described above, we expected IA to be impaired in individuals with ASD. Therefore, the following hypotheses were tested:

1. The ASD group will make more errors than the TD group when judging the ending in the IA, but not the PC condition.
2. In accordance with previous studies, the P3-like component in posterior electrodes should be stronger for IA than PC in the TD group. If IA is based in contextual integration processes, and if these are impaired in individuals with ASD, this effect should not be found in the ASD group.
3. Incongruous endings should cause a P3-like component in IA and PC conditions in TD, signifying deliberate contextual integration. If the ability to predict the ending in the IA condition is limited in individuals with ASD, this effect should not be observed in the IA condition in the ASD group.
4. Finally, incongruous endings should cause a stronger late (approximately 700–1200 ms) ongoing positive component in both conditions in TD. If impaired IA in individuals with ASD is rooted in inflexible integration of expectation violations, the amplitude of this component should be stronger in the ASD group.

## Methods

### Participants

EEG data was collected from a total of 26 participants with ASD and 24 TD controls. The final sample of participants whose data could be included in the analysis consisted of 20 children and adolescents with ASD and 21, age, IQ and sex matched TD (see Table 1). Participants were recruited via the Department of Child and Adolescent Psychiatry, Psychosomatics and Psychotherapy of the University Hospital, Goethe-University Frankfurt, Germany. The research project was carried out in accordance with the Declaration of Helsinki (World Medical Association, 2013) and approved by the local ethics committee of the Medical Faculty of the Goethe University Frankfurt am Main. Written informed consent was obtained from caregivers, written informed assent was obtained from the participants. For ASD and TD groups, inclusion criteria were an IQ ≥ 70, age between 10 and 17 years, and normal or corrected-to-normal vision. The age range of the ASD group was 10;3–17;8 years, that of the TD group was 13;1–17;7 years. Additionally, participants in the ASD group had to have an expert ICD-10 diagnosis of autism, atypical autism or Asperger Syndrome based on the Autism Diagnostic Observation Schedule, second version (ADOS-2; Lord et al., 2015; German version: Poustka et al., 2015) and the Autism Diagnostic Interview-Revised (ADI-R; Lord et al., 1994; German version: Bölte et al., 2006). Exclusion criteria for both groups were any neurological conditions, preterm birth (birth weight ≤ 2000 g or born < 32nd week of pregnancy), psychotropic medication (with the exception of stimulants). Prior to the examination, immediate release stimulants were stopped for 24 h, sustained-release stimulants for 48 h. All participants were screened for any current or past psychiatric disorders and excluded if they had a suspected or confirmed diagnosis of Depressive, Bipolar-, Anxiety-, Obsessive–Compulsive-, Tic-, Oppositional-Defiant-, Conduct- or Substance Abuse Disorder or Schizophrenia using the Child Behavior Checklist (Achenbach, 1991). TD with Attention Deficit (Hyperactivity) Disorder (ADD, ADHD) were also excluded. However, participants in the ASD group with comorbid AD(H)D were not excluded due to the high rate of comorbidity of AD(H)D and ASD of around 28% (for a review, see Lai et al., 2019). Here, N = 4 participants showed the inattentive type, N = 2 the hyperactive/impulsive type and N = 2 the combined type.

**Table 1:** Sample description

Sample description	TD (N = 21)	ASD (N = 20)	t (df)	p
Age				
Mean (SD)	15.89 (1.31)	15.53 (2.12)	0.6761 (39)	0.503
IQ				
Mean (SD)	102.90 (7.75)	98.25 (12.09)	1.4752 (39)	0.148
Handedness				
Left	2	3		0.663^a^
Right	19	17		
Gender				
Male	18	17		1.000^a^
Female	3	3		
ADOS CSS				
Mean (SD)	n/a	5.85 (2.70)		

*ASD* autism spectrum disorder, *TD* typically developing, *IQ* intelligence quotient, *ADOS* autism diagnostic observation schedule, *CSS* calibrated severity score

^a^Significance according to Fisher’s Exact Test (assumptions for Chi-square test were violated)

IQ was measured using the Vocabulary and Picture Completion subscales of the third editions of the Wechsler Intelligence Scale for Children (Wechsler, 1991; German version: Tewes & Rossmann, 2002)/Adults, Wechsler, 1997; German version: Tewes et al., 2001), respectively. This abbreviated version was applied because it covers both verbal and performance IQ, while keeping testing time at a minimum. The Edinburgh Handedness Inventory (EHI; Oldfield, 1971) was used to determine participants’ handedness.

### Materials and Design

#### Procedures

**Fig. 1:**
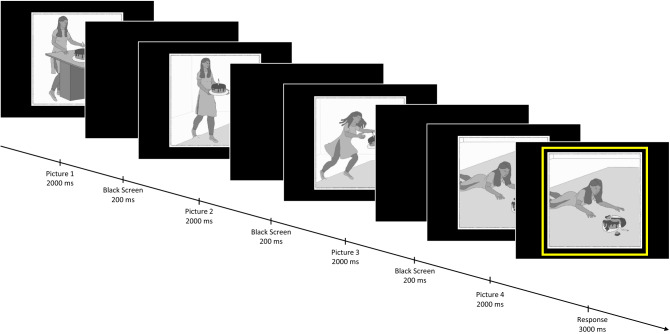
Timing diagram of one trial. Images were shown sequentially. Participants were instructed to indicate whether the ending was correct or incorrect, but withhold their response until the yellow frame appeared around the fourth image

**Fig. 2:**
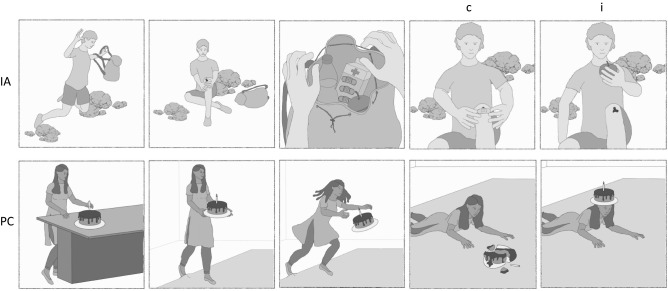
Example comics for the Intention Attribution (*IA*) and Physical Causality (*PC*) conditions with congruent (*c*) and incongruent (*i*) endings

Stimuli consisted of gray-tone comic strips. Comics were taken or adapted from previous studies (Brunet et al., 2000; Murdaugh et al., 2014; Vistoli et al., 2011, 2015; Walter et al., 2004) with the authors’ permission. Additional comics were created by authors MS and HM using a Wacom Intuous graph pad and pen (Version CTH-690) and Clip Studio Paint Pro software (Version 1.7.3). All comics included one human protagonist, in order to increase similarity between PC and IA conditions. Each comic consisted of four images that were presented sequentially. Each picture was presented for 2000 ms, followed by a black screen (200 ms). The first two images served to establish context, so that when the 3rd image was presented, participants were able to infer an ending to the story and predict what would happen in the 4th picture. Once the 4th image had been presented for 2000 ms, a yellow frame appeared around the image for an additional 3000 ms, during which time participants were instructed to give their response. This was done to prevent response-related motion artifacts during the initial observation of the 4th image, enabling us to evaluate ERPs during this timeframe. Inter-trial-intervals (ITIs) were jittered between 1400 and 1600 ms. For a timing diagram, see Fig. 1. Comics depicted either a physical causality (PC; e.g. a falling object) or an intention (intention attribution; IA; e.g. fetching a ladder to reach something). Comics had congruous (-c) or incongruous (-i) endings (e.g. incongruous: an object floating up for PC or a person fetching an unexpected tool for IA). For examples of the stimuli and different conditions, see Fig. 2. Participants were instructed to observe the comics, attend to the depicted story and decide whether the ending depicted in the 4th image was the logical consequence of the three previous pictures. Responses were given using left and right mouse buttons; the left button indicated a congruous ending, the right button indicated an incongruous ending.

The task was part of a test battery of four tasks in total, which all participants completed in random order. Each participant completed two practice trials before the experiment in order to ensure that they had understood the instructions and were familiar with the correct allocation of the response buttons. The experiment consisted of 60 trials in total. 30 trials depicted IA stories, 30 depicted PC; half of each had congruous and half had incongruous endings, resulting in 15 IA-c, 15 IA-i, 15 PC-c and 15 PC-i trials. The order of presentation was pseudorandomized, with the constraint that the same condition was never shown more than twice in a row. After 20 and 40 trials, there was a break that participants could end with a button-press. Total duration of the task was approximately 16 min.

Presentation of images was implemented in the Presentation software (Neurobehavioral Systems, Albany, CA, USA). Images were 17.5 by 17.5 cm and presented on a black background. The distance between participants and the monitor was 80 cm, resulting in a visual angle of 12.5°.

#### Pilot Study

As many previous studies suggested that the ERPs of interest in this study might be weaker due to age effects, the paradigm was tested in a sample of N = 6 children (mean age = 13.5 ± 1.71). The sample consisted of two males and four females; one participant did not show any psychiatric or neurodevelopmental disorder, five were suffering from major depressive disorder. In the pilot study, the P3-like component during the 3rd image was significantly greater for IA than PC (F(1,6) = 7.873; *p* = 0.031; η^2^ = 0.567), demonstrating that the ERP was present even at a younger age. ERPs during the 4th image were not examined in the pilot-study.

#### EEG-Data Acquisition

64 channel caps in an extended 10–20 international layout (10–10 system; Easycap GmbH, Herrsching, Germany) with sintered Ag/AgCl electrodes were used to record EEG-data. FCz was used as recording reference and impedances were kept below 10 kΩ for all electrodes. Brain Vision Recorder software and Brain Vision MR-Plus amplifiers (Brain Products GmbH, Munich, Germany) were used to record data, and an online anti-aliasing low pass filter with 0.01 Hz low cut-off and 250 Hz high cut-off and a sampling rate of 1000 Hz was applied. An electrode placed 1 cm below the right eye was used to record eye movements and blinks.

### Analysis

#### Behavioral Data

To examine task performance, hit rate (HR) and false alarms (FA) were z-transformed using ${(x}_{i}-\mu )/\sigma$. In order to determine sensitivity, d′ was calculated using ${z}_{HR}-{z}_{FA}$.

#### EEG-Data

EEG-data were analyzed in Brain Vision Analyzer 2.2 software (Brain Products GmbH, Munich, Germany). Data were down-sampled to a sampling-rate of 500 Hz and re-referenced to average reference (AR). Please note that a linked mastoid reference (LMR) is commonly used for analyzing the P3-component, as it has the potential to enhance its amplitude (i.e. Dien, 1998). However, we chose to use the average reference (AR) for our data. Despite low impedances, visual inspection of the data for AR vs. LMR showed artifact contamination of the signal from electrodes TP9 and TP10 in approximately 1/4 of participants. Therefore, a LMR based on TP9 and TP10 could not be applied. Furthermore, while LMR is most commonly used in P3-research, this has recently been debated, as LMR has been shown to alter signal distributions (e.g. Liu et al., 2015). Additionally, AR is more commonly used to examine the late frontal ERP component associated with the processing of expectation violations, which was also a focus of our study. A high-pass filter of 0.1 Hz (eighth order Butterworth zero phase shift filter) was applied. Data were manually cleaned from gross artifacts (e.g. muscle artifacts) and an independent component analysis (ICA) was run using the “infomax” algorithm in Brain Vision Analyzer and artifact components, such as eye blinks, line noise or muscle tension were removed.

**Fig. 3:**
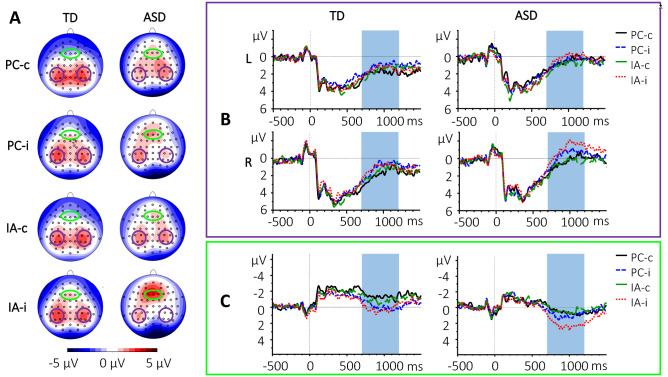
Event related potentials during 700–1200 ms after onset of the 4th image. **A** Topographies in the typically developing (TD) and Autism Spectrum Disorder (ASD) groups for intention attribution (IA) and physical causality (PC) and correct (-c) and incorrect (-i) endings. The parietal clusters are marked in purple, the frontal cluster is marked in green. **B** Grand average ERP waveforms for left (L) and right (R) parietal clusters in TD and ASD for both conditions and endings. The time of interest is marked in blue. **C** Grand average ERP waveforms for the frontal cluster in TD and ASD groups for both conditions and endings. The time of interest is marked in blue

Data were segmented into epochs of 2000 ms (− 500 to 1500 ms around stimulus-onset) and a low-pass filter of 30 Hz (eighth order Butterworth zero phase shift filter) was applied. Data were baseline-corrected using the 500 ms before stimulus-onset as baseline. This baseline window was chosen because, as evident in Fig. 3B and C, the onset of the black screen (baseline) between the task-related images causes what we believe to be a P1/N1-component complex associated with onset of a new visual stimulus (for a review, see Correa et al., 2006). This P1/N1 component was present over almost the entire inter-stimulus interval (ISI) in our data. As the purpose of baseline correction is to establish a meaningful reference point to which the ERP-components of interest can be compared (Urbach & Kutas, 2006), the last 300 ms of the previous image were included in the baseline in addition to the ISI. As images were presented for 2000 ms and no action was required during the presentation of the first three images, these additional 300 ms demonstrated very little ERP activation, serving to “neutralize” the P1/N1 complex elicited during the ISI. Segments containing remaining artifacts were rejected semi-automatically using the following criteria: Maximal allowed voltage step: 50 µV/ms; maximal allowed difference of values in intervals: 200 µV; lowest allowed activity in intervals: 0.5 µV. Only data of participants of which at least 2/3 of the trials in each category were viable were included for further analyses. The mean number of the remaining trials per group and condition can be found in Supplement 1. Averages were computed for the different categories: AI and PC for the 3rd image, further divided by ending (congruous/incongruous; c/i) for the 4th image.

Electrodes and timeframes of interest were determined based on literature (Vistoli et al., 2011, 2015) as well as visual inspection of grand average data.

For the 3rd image, bilateral parietal electrode clusters were formed (P3, P5, PO3, PO7 for a left and P4, P6, PO4, PO8 for a right parietal cluster). Mean amplitude (μV) between 200 and 700 ms after stimulus onset was exported to examine the P3-like component, as described in Vistoli et al. (2011, 2015).

For the 4th image, mean amplitude of the same clusters and timeframe were exported. Furthermore, mean amplitude (μV) for a frontal cluster (F1, F2, Fz) between 700 and 1200 ms after onset of the 4th image were exported to examine the effect of incongruous endings. Following further visual inspection of the grand average waveforms and topographies, a negative deflection between 700 and 1200 ms at parietal electrodes could also be observed. Therefore, mean amplitude (μV) of bilateral parietal clusters (P3, P5, CP3, CP5 for the left and P4, P6, CP4, CP6 for the right cluster) were also exported.

#### Statistical Analysis

For behavioral data, a 2 × 2 ANCOVA with the factors Group (ASD vs. TD) and Condition (IA vs. PC) was calculated in SPSS (Version 26) with d’-values as the dependent variable. To control for the effects of IQ, it was added as a covariate to the model. We did not compare RT, as the responses were standardized and artificially delayed due to the stimulus adaptation for the EEG study.

All statistical analyses of ERP data were conducted using Statistica (version 13). For the 3rd image, a mixed-model ANCOVA with the between-factor Group (ASD vs. TD) and the within-factors Condition (AI vs. PC) and Hemisphere (left vs. right parietal cluster) was calculated. IQ-scores were added to the model as a covariate.

For all ERP analyses of the 4th image, the within-factors Condition (AI vs. PC), Ending (congruous vs. incongruous) and Hemisphere (left vs. right parietal cluster) were used to calculate a mixed-model ANCOVA with IQ as a covariate and between-factor Group (ASD vs. TD).

Normality of residuals, sphericity and homoscedasticity of the data were confirmed prior to statistical analysis. Bonferroni-corrected post hoc tests were applied to any interaction effects to assess the direction of the effect.

## Results

### Behavioral Data

Sensitivity for distinguishing congruous from incongruous endings, independent of condition did not differ between groups (*F*(1,38) = 2.418, *p* = 0.13, $\eta_{{\text{p}}}^{2}$ = 0.06; Table 2). No main effect of Condition (*F*(1,38) = 0.784, *p* = 0.38; $\eta_{{\text{p}}}^{2}$ = 0.02) or interaction effect of Condition ×  Group (*F*(1,38) = 0.05, *p* = 0.82, $\eta_{{\text{p}}}^{2}$ = 0.00) was observed.

**Table 2:** Behavioral data

Behavioral data	TD (N = 21)	ASD (N = 20)
Sensitivity (d′)		
IA	0.54 (1.54)	− 0.41 (2.26)
Mean (SD)		
PC	0.41 (1.31)	− 0.28 (1.69)
Mean (SD)		

Effects	F (df)	ps	η^2^
Group	2.418 (38)	0.128	0.06
Condition	0.784 (38)	0.382	0.02
Group × condition	0.050 (38)	0.824	0.00

*ASD* autism spectrum disorder, *TD* typically developing, *IA* intention attribution, *PC* physical causality

### Event-Related Potentials

#### Third Image

No significant main or interaction effects were found for mean amplitude (μV) in either one of the parietal electrode clusters during the 200–600 ms time window during the observation of the 3rd image. For descriptive statistics, multivariate tests, as well as the topographies and grand average of the ERP, see Supplement 2A–2D.

Despite the positive results of the pilot study, we did want to examine the effect of age. However, age could not be included as a covariate in the model as it did not fulfill the assumption of homogeneity of slopes. To determine whether the lack of a main effect of condition was due to age, a Pearson-correlation was calculated for the difference between mean amplitude of PC and IA conditions and age. Only small effects were observed in the ASD (*r* = 0.220, *p* = 0.175) and in the TD group (*r* = 0.368, *p* = 0.050).

#### Fourth Image

No significant main or interaction effects were found for mean amplitude in either one of the electrode clusters during the 200–600 ms time window during the observation of the 4th image. For descriptive statistics, results of multivariate tests, as well as the topography and grand average of the ERP, see Supplement 3A—3D.

During the 700–1200 ms time frame, the following effects were found for mean amplitude in the parietal clusters: Group × Ending (*F*(1,38) = 4.67, *p* = 0.004, $\eta_{{\text{p}}}^{2}$ = 0.11) and Group × Condition × Ending (*F*(1,38) = 4.430, p = 0.042, $\eta_{{\text{p}}}^{2}$ = 0.10). Post-hoc tests revealed that in the TD group, congruous and incongruous endings caused no significant difference in activation in the parietal clusters (*M*_congruous_ = 1.81 ± 1.01 µV; *M*_incongruous_ = 1.84 ± 0.92 µV; *p* = 1.0), but incongruous endings caused a significantly stronger negative component than congruous endings in the ASD group (*M*_congruous_ = 1.81 ± 1.43 µV; *M*_incongruous_ = 0.64 ± 0.95 µV; *p* = 0.03; see also Fig. 3A and B). The three-way interaction showed that in the ASD group the mean amplitude was larger for incongruous compared to congruous endings in the IA condition (*p* = 0.01). The amplitude for incongruous IA endings in the ASD group was also significantly larger than the amplitudes for all conditions in the TD group (IA-c_TD_ *p* = 0.01; IA-i_TD_ *p* = 0.01; PC-c_TD_ *p* = 0.03; PC-i_TD_ *p* = 0.03). For descriptive statistics, multivariate tests, as well as significant post-hoc tests see Supplement 4A–4D.

This three-way interaction of Group ×  Condition × Ending (*F*(1,38) = 5.326, *p* = 0.027, $\eta_{{\text{p}}}^{2}$ = 0.12) was also found in the frontal cluster during the 700–1200 ms timeframe, where incongruous endings in the IA condition again elicited significantly larger amplitudes than congruous endings in both conditions in both groups (IA-c_ASD_ *p* < 0.01; PC-c_ASD_ *p* = 0.01; IA-c_TD_ *p* = 0.01; IA-c_TD_ *p* < 0.01). Furthermore, congruous and incongruous endings in the PC condition caused a significant difference in activation in the TD (M_PC-c_ =  − 0.93 ± 1.78 µV; M_PC-i_ = 0.51 ± 2.02 µV; *p* = 0.03), but not the ASD group (M_PC-c_ = 0.81 ± 1.93 µV; M_PC-i_ = 1.26 ± 2.07 µV; *p* = 1.0; see also Fig. 3A and C). For descriptive statistics, multivariate tests, as well as significant post-hoc tests see Supplement 5A–C.

## Discussion

The aim of the present study was to expand findings of impaired IA and underlying neural processes in ASD. Specifically, borrowing from PCT, we hypothesized impaired IA in individuals with ASD would be associated with aberrant contextual updating processes and/or inflexible processing of expectation violations. Results revealed no IA abnormalities in the ASD group on the behavioral level, while ERP evidence suggested that processing abnormalities associated with IA are mainly found when incongruous information needs to be processed.

### Behavioral Measures

There was no sign of impaired IA in the ASD group on a behavioral level. There are three studies that have previously examined the comic strip paradigm in participants with ASD, with mixed behavioral results: two found that individuals with ASD made more errors in the IA-condition, but not the PC-condition (Kana et al., 2014; Murdaugh et al., 2014), one found individuals with ASD generally made more errors in both conditions (Ciaramidaro et al., 2015). As mean age of participants in the current study was lower than in in these previous studies, the good performance of ASD participants cannot be attributed to age effects. Furthermore, average IQ was similar to those in the other studies. One potential reason that no effect of group or an interaction with condition was found here could be a slight difference in the task design: all three previous studies presented participants with three different endings, from which they had to choose the correct one, whereas in this study, participants were shown one ending and had to indicate whether it was right or wrong. This design was chosen in order to enable ERP analysis for congruous and incongruous endings. It may, however, have altered task difficulty. Future studies should consider this in their task design.

### Third Image

No significant effects were found for the timeframe of 200–600 ms after onset of the 3rd image. Vistoli et al., (2011, 2015) found a significantly stronger P3-like component for IA compared to PC for this time window in TD adults. While the ERPs in the present study show a similar effect in both groups when visually examined (see Supplement 4A/4B), suggesting that the stimulus material used in the present study was appropriate, this effect did not reach significance. The effect of condition may have been obscured by the higher variance in neural activation in the ASD group (see Supplement 2A). Furthermore, the difference between PC and IA was correlated with age. As our sample was younger than that of the previous ERP studies described (Vistoli et al., 2011, 2015), this suggests that the lack of a main effect of condition might be due to our younger sample. Despite the promising results of the pilot-study, it is possible that the difference in activation between IA and PC was obscured by age effects. This is in line with findings that demonstrate ongoing brain maturation processes in areas associated with social cognition, including the TPJ (Blakemore, 2008, 2012; Choudhury et al., 2008; Dumontheil, 2015; Mills et al., 2014). In particular, the fMRI study contrasting IA and PC sentences in adolescents found that the difference in activation in parietal areas was lower in adolescents than adults (Blakemore et al., 2007). These findings suggest that the effect of interest is smaller in adolescents than it is in adults, and that our sample size was too small to detect it. Furthermore, while females typically perform better in social cognition tasks throughout their lifetime (Gur et al., 2012; Williams et al., 2009), these sex differences are most pronounced in mid-adolescence (Gur et al., 2012) and are analogous to findings that report earlier brain maturation in females (Hills & Byrne, 2010). ASD disproportionately affects males (Loomes et al., 2017), which is reflected in the high ratio of male participants in the current sample. Including mostly male participants may have further reduced our ability to find the predicted P3-like component. Another contributing factor may have been the relatively small sample size. Future studies should address these concerns in a larger, older sample with a higher ratio of female participants.

We expected a group by condition interaction effect, i.e. a stronger P3-like component in IA than PC for TD, but not ASD participants. This component reflects activation of the TPJ associated with contextual updating (Friedman et al., 2001; Ibanez et al., 2012), which we expected to be impaired in individuals with ASD. This was not observable in our data. The lack of significant findings may again be related to the larger variance in mean amplitude for this component observed in the ASD group, especially in the IA condition. The results do, however, also indicate that the integration of information from the first two images and inference of intention were not as severely affected in the ASD group as expected. Support for this interpretation comes from the observation of intact behavioral task performance. Also, given the increased amplitude of the component for incongruous endings in the IA condition during the 4th image, it can be inferred that ASD individuals must have predicted the ending correctly in order to show a differential response to an incongruous one.

### Fourth Image

Our study is the first to analyze ERP components related to neural processing of congruous and incongruous endings in the comic strip paradigm in participants with ASD. We expected to observe a P3-like component, similar to those found for the processing of picture 3 (Vistoli et al., 2015). We expected this component to be larger for incongruous endings, representing a process of contextual integration, which should take place when an incongruous ending is detected. We further hypothesized that this effect would be observed for both conditions in the TD, but only for the PC condition in the ASD group. A component that matched a parietal mid-latency positivity (200–600 ms) was observable in our data. However, subsequent analysis revealed that this component was neither sensitive to congruous vs. incongruous endings, nor did it indicate any between-group differences in the processing of endings at this stage. The lack of condition effects for this component may be related to stimulus complexity. Previous ERP studies examining congruous vs. incongruous endings used stories (e.g. van Duynslaeger et al., 2007, 2008), i.e. the incongruous ending consisted of a single word, whereas the current study used quite complex visual stimuli. Therefore, contextual updating may be required in all conditions, not only in IA in this paradigm. Furthermore, incongruency may take longer to be detected in complex stimuli: A recent study examining the neurophysiological processes associated with visual awareness found evidence of a temporal unfolding of ERP markers: early components in this study reflected initial perception, while later components correlated with the conscious experience of non-perceptual information (Derda et al., 2019). It is, therefore, likely that, across groups and conditions, the 200–600 ms timeframe was used for initial perception and contextual integration (as supported by the P3-like component resembling the one in the 3rd image, see Supplement 3C), and the awareness as to whether it was congruous or incongruous occurred later. Interestingly, this was observed for both the ASD and the TD group. Taking into account that behavioral results also indicated no differences in task performance, our findings suggest that the processing of complex visual stimuli and contextual integration is intact in individuals with ASD.

In the later time window, during which we hypothesized the conscious processing of congruous and incongruous endings would take place, significant differences between the groups emerged.

In parietal areas, the ASD group showed a stronger negative deflection for incongruous endings. This effect was particularly strong in the IA condition, suggesting that the ASD group was, in fact, able to predict the congruous ending of the stories, even when the prediction was based on IA. Furthermore, these results suggest a more effortful (i.e. stronger) processing of expectation violations in the ASD group. Unfortunately, there are only few studies that have systematically examined late ERPs, making it difficult to interpret results in this timeframe. One relevant study looked at a similar component, the late posterior negative slow wave (LPN). The LPN is often reported in studies of memory-retrieval and most often associated with tasks requiring complex memory-retrieval (Johansson & Mecklinger, 2003). A cautious interpretation of the stronger negative component in parietal electrodes for incongruous endings (in the IA condition) could be that it signifies a deliberate effort of the ASD group to retrieve contextual information from the previous images and integrate it with the incongruous information.

A similar pattern of effects was found for a frontal electrode cluster in the same late time window. Here, incongruous endings in the IA condition caused a significantly larger positive component in the ASD group. This late positive component bears resemblance to the P600 component often found for expectation violation in language- or sentence processing: a late and extended positive component with no clear peak between 500 and 900 ms after stimulus onset in centro-parietal electrodes. Several studies have also found a distinctly frontal P600 when the end of a sentence or discourse is unexpected in the given context (DeLong et al., 2014; Federmeier et al., 2007; Otten & van Berkum, 2008). Furthermore, a P600 effect has also been demonstrated for arithmetic (Núñez-Peña & Honrubia-Serrano, 2004) as well as harmonic rule violations (Patel et al., 1998) and visual oddball detection (Sassenhagen & Fiebach, 2019). This suggests that this component is likely not limited to the violation of syntactic rules, but can be seen as general index of rule-violations. A study by van der Cruyssen et al. (2009) found that this component was stronger when participants deliberately thought about the goal of an observed action. This component could, therefore, signify deliberate processing of the observed expectation violation. Although the clinical presentation indicates that expectation violations are likely aberrantly processed in ASD, there are only few studies that have explicitly examined it. One study examining behavioral and pupillometric data in participants with ASD using a probabilistic associative learning task reported decreased distinction between expected and unexpected outcomes (Lawson et al., 2017). In contrast, an MEG-study compared congruous and incongruous endings of sentences in ASD participants and found that incongruous endings elicited long-lasting (600–1000 ms) gamma-oscillations in individuals with ASD, which the authors interpreted as a strategy to resolve ambiguity (Braeutigam et al., 2008)—a result that seems more in line with the present findings.

The observed increased neural processing of stimuli that represent the violation of expectation is also relevant in the context of PCT, a theory that proposes the aberrant generation of predictions and altered processing of incoming information as a possible underlying mechanism of ASD (see e.g. Pellicano & Burr, 2012; van de Cruys et al., 2014). There is disagreement about which part of the predictive process is affected in ASD. Some authors posit that the influence of prior knowledge (PK) is reduced, i.e. there is a reduced reliance on previously generated mental models of the world (Pellicano & Burr, 2012). Others suggest an increased influence of the prediction error (PE; Brock, 2012; van de Cruys et al., 2014), i.e. if a PE occurs, mental models are updated too quickly based on this error and are less reliable as a result. In this context, our findings might point towards an increased influence of the PE (van de Cruys et al., 2014) that leads to a more effortful (i.e. stronger) processing of expectation violations. In fact, this variant of PCT also predicts that expectation violations in social stimuli should be much more difficult to process for individuals with ASD, as they are more complex (van de Cruys et al., 2014)—explaining why incongruous endings in the IA condition lead to particularly large amplitudes of the P600 in the ASD group in the present study. While the present study did not explicitly examine PCT, results suggest that it would be worthwhile to apply this framework to social cognitive abilities in ASD. A recent review examining all computational neural network models of ASD also came to the conclusion that PCT is one of the most promising frameworks to explain all ASD symptoms (Lanillos et al., 2020).

Studies on expectation violation and contextual integration in participants with ASD remain scarce. Future studies should examine these topics in order to elucidate underlying processes, and, if possible, examine the role of PK and PE in these processes according to the PCT framework.

## Summary and Conclusion

In summary, results suggest that individuals with ASD were just as capable as TD of predicting and identifying congruous endings based on IA. Both on a behavioral level and in early ERPs that indicate contextual updating, no significant differences between groups or interaction of Group and Condition were found. However, future studies should apply this paradigm in older participants (with and without ASD) to examine whether any potential group effects might have been obscured by age, due to ongoing maturation of areas such as the TPJ (Blakemore, 2008, 2012; Choudhury et al., 2008; Dumontheil, 2015; Mills et al., 2014).

Later ERPs, likely signifying a more deliberate and conscious evaluation of endings, show a significant difference in parietal electrodes between the TD and ASD groups for incongruous endings, especially in the IA condition. This might reflect a conscious attempt to retrieve information from previous images and integrate it with the current incoming information. A frontal component also shows a more effortful processing of incongruous endings in the IA condition in the ASD group: this component appears to be associated with expectation violations, especially for social stimuli encompassing human actions. This could, again, reflect a deliberate cognitive effort in individuals with ASD to make sense of the observed behavior. In line with previous findings (Braeutigam et al., 2008), this suggest that individuals with ASD struggle to process expectation violations, as proposed by the PCT framework (van de Cruys et al., 2014). Future studies should examine the relationship of expectation violation and impaired IA in individuals with ASD more closely.

## Supplementary Information

Below is the link to the electronic supplementary material.

Supplementary file1 (PDF 968 kb)
