# Supplementary material for: Intention Attribution in Children and Adolescents with Autism Spectrum Disorder: An EEG Study
Source: J Autism Dev Disord. 2021 Dec 2;53(4):1431–43. doi: 10.1007/s10803-021-05358-1 (PMC10066121; doi:10.1007/s10803-021-05358-1)
Supplement: Supplementary file 1 — Supplementary file1 (PDF 968 kb) [file 10803_2021_5358_MOESM1_ESM.pdf]

Supplementary Material

Article Title: "Intention Attribution in Youth with Autism Spectrum Disorder: an EEG Study"

Journal of Autism and Developmental Disorders

Magdalena Schütz, Sara Boxhoorn, Andreas Mühlherr, Hannah Mössinger, Christine M. Freitag[#] & Christina Luckhardt[#]

Corresponding author: Magdalena Schütz

Department of Child and Adolescent Psychiatry, Psychosomatics and Psychotherapy, Autism Research and Intervention Center of Excellence, University Hospital Frankfurt, Goethe University, Frankfurt am Main, Germany

E-Mail: Magdalena.Schuetz@kgu.de

Supplement 1: Mean (±SD) number of trials remaining for analysis for each group and condition

	3rd Image		*4th Image*			
	Intention Attribution	***Physical Causality***	***Intention Attribution***		***Physical Causality***	
			Congruous	***Incongruous***	***Congruous***	***Incongruous***
ASD	24.60 (±2.41)	24.80 (±3.33)	12.70 (±1.56)	12.35 (±1.90)	12.70 (±1.98)	13.05 (±1.79)
TD	25.75 (±2.77)	27.25 (±2.24)	13.50 (±1.64)	13.50 (±1.40)	13.50 (±1.61)	13.90 (±1.07)

Note. ASD = Autism Spectrum Disorder; TD = Typically Developing. Group differences reached significance for the Physical Causality condition during the 3rd image ($T(1,38) = -2.762$; $p = 0.01$).

Supplement 2A: Mean (±SD) area (µV*ms), 200 – 600 ms, parietal clusters, 3rd image

	Intention Attribution		***Physical Causality***	
Hemisphere	***left***	***right***	***left***	***right***
ASD	4.03 (±3.76)	4.09 (±4.58)	2.36 (±2.78)	3.09 (±3.62)
TD	3.60 (±2.57)	3.78 (±2.56)	2.61(±1.70)	3.29 (±2.94)

Note. ASD = Autism Spectrum Disorder; TD = Typically Developing

Supplement 2B: Multivariate tests, 200 – 600 ms, parietal clusters, 3rd image

	F (1, 38)	***p***	***η²***
Condition	0.827	0.369	0.021
Condition * IQ	0.248	0.621	0.006
Condition * Group	1.761	0.192	0.044
Hemisphere	0.134	0.716	0.004
Hemisphere * IQ	0.043	0.836	0.001
Hemisphere * Group	0.067	0.797	0.002
Condition * Hemisphere	1.404	0.243	0.036
Condition * Hemisphere * IQ	0.825	0.367	0.021
Condition * Hemisphere * Group	0.055	0.816	0.001
IQ	3.328	0.076	0.081
Group	0.153	0.698	0.004

Supplement 2C: Topographies and average ERP waveforms for the 3rd image (200 – 600 ms).

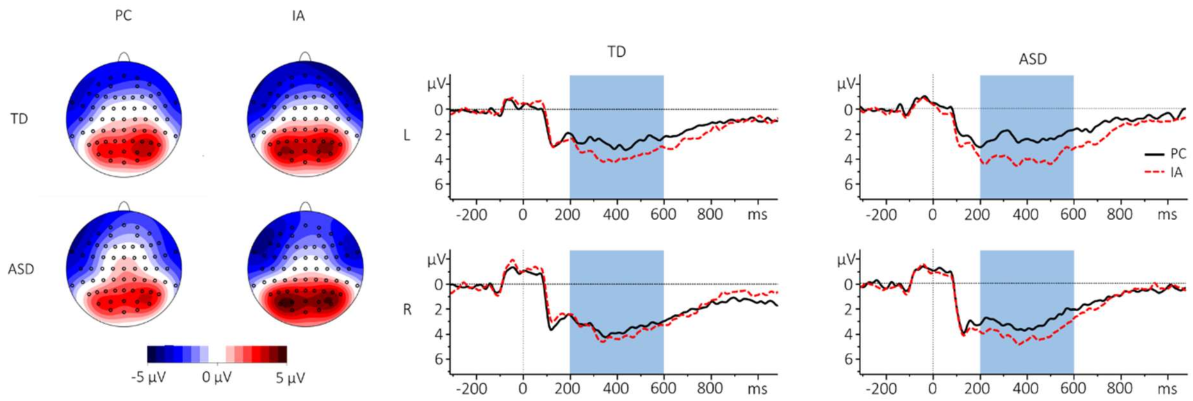

Note. Topographies for the 3rd image in the typically developing (TD) and Autism Spectrum Disorder (ASD) groups for physical causality (PC) and intention attribution (IA) during 200 – 600 ms after onset of the 3rd image. Grand average ERP waveforms show left (L) and right (R) parietal clusters for both groups and conditions. The time of interest is marked in blue.

Supplement 3A: Mean (±SD) area (µV*ms), 200 – 600 ms, parietal clusters, 4th image

	Intention Attribution				***Physical Causality***			
Ending	***Congruous***		***Incongruous***		***Congruous***		***Incongruous***	
Hemisphere	***left***	***right***	***left***	***right***	***left***	***right***	***left***	***right***
ASD	3.74	3.90	3.54	3.52	3.05	3.60	3.37	3.70
	(±4.98)	(±4.98)	(±4.32)	(±4.99)	(±3.77)	(±4.86)	(±3.33)	(±4.19)
TD	3.73	4.60	3.56	4.05	3.51	4.47	2.99	4.19
	(±2.65)	(±2.86)	(±2.70)	(±2.92)	(±2.59)	(±3.11)	(±2.50)	(±2.92)

Note. ASD = Autism Spectrum Disorder; TD = Typically Developing

Supplement 3B: Multivariate tests, 200 – 600 ms, parietal clusters, 4th image

	F (1, 38)	*p*	*η²*
Condition	5.70	0.455	0.015
Condition * IQ	0.757	0.390	0.020
Condition * Group	0.095	0.760	0.002
Ending	0.475	0.495	0.012
Ending * IQ	0.668	0.419	0.017
Ending * Group	0.627	0.433	0.016
Hemisphere	0.595	0.445	0.015
Hemisphere * IQ	0.358	0.553	0.009
Hemisphere * Group	1.230	0.274	0.031
Condition * Ending	0.183	0.671	0.005
Condition * Ending * IQ	0.248	0.671	0.005
Condition * Ending * Group	0.847	0.363	0.022
Condition * Hemisphere	2,439	0.127	0.060
Condition * Hemisphere * IQ	1.802	0.187	0.045
Condition * Hemisphere * Group	0.137	0.713	0.004
Ending * Hemisphere	0.038	0.846	0.001
Ending * Hemisphere * IQ	0.067	0.797	0.002
Ending * Hemisphere * Group	0.131	0.719	0.003
Condition * Ending * Hemisphere	1.156	0.289	0.030
Condition * Ending * Hemisphere * IQ	0.980	0.328	0.025
Condition * Ending * Hemisphere * Group	1.556	0.220	0.039
IQ	2.900	0.097	0.071
Group	0.511	0.479	0.013

Supplement 3C. Topographies and average ERP waveforms for the 4th image (200 – 600 ms)

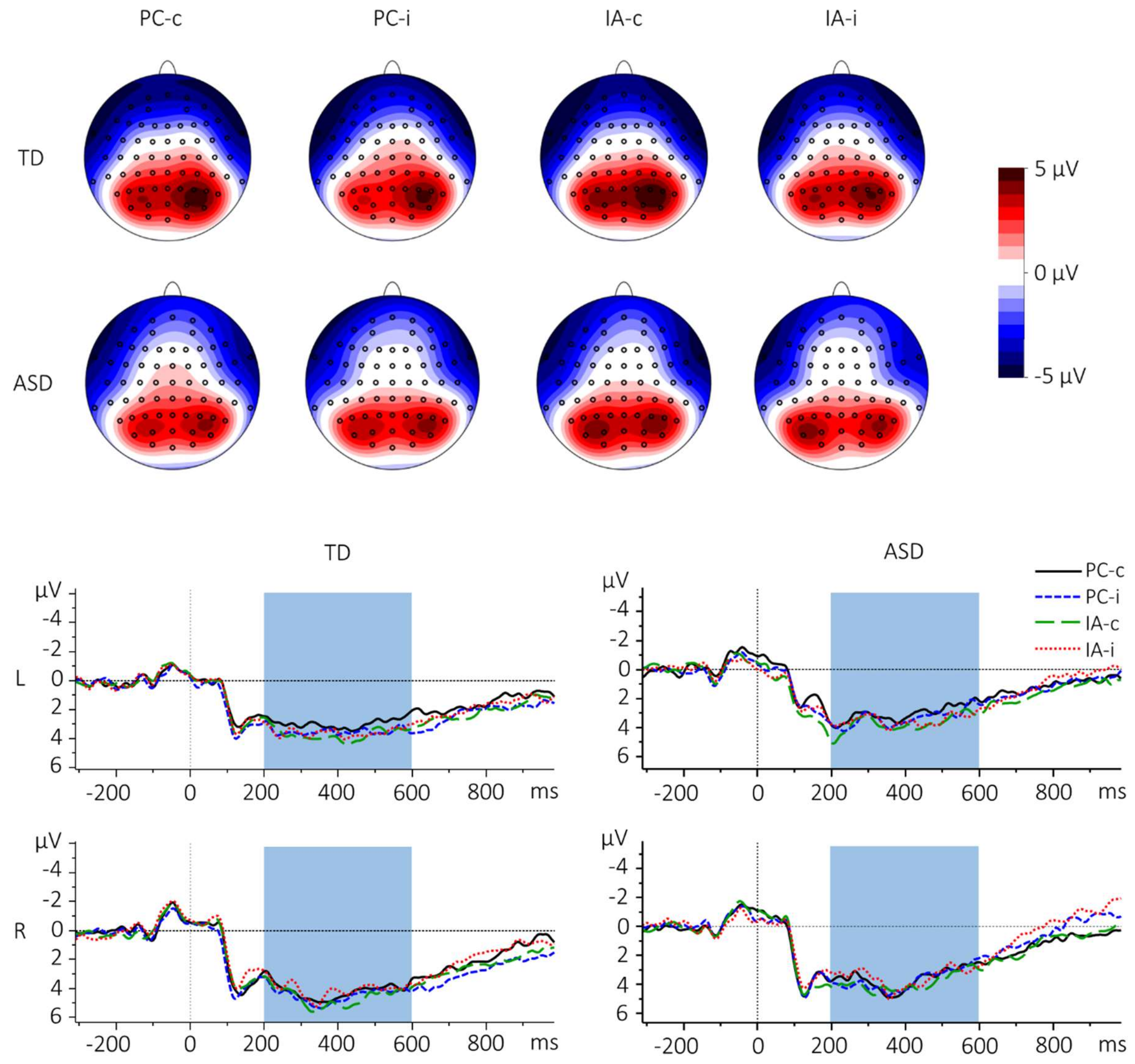

Note. Topographies in the typically developing (TD) and Autism Spectrum Disorder (ASD) groups for physical causality (PC) and intention attribution (IA) and correct (c) and incorrect (i) endings during 200 – 600 ms after onset of the 4th image. Grand average ERP waveforms show left (L) and right (R) parietal clusters for both groups, conditions and endings endings during 200 – 600 ms after onset of the 4th image. The time of interest is marked in blue.

Supplement 4A: Mean (±SD) area (µV*ms), 700 – 1200 ms, parietal clusters, 4th image

	Intention Attribution				*Physical Causality*			
Ending	***Congruous***		***Incongruous***		***Congruous***		***Incongruous***	
Hemisphere	***left***	***right***	***left***	***right***	***left***	***right***	***left***	***right***
ASD	1.33	1.20	0.99	-0.22	0.97	1.19	1.31	0.47
	(±1.51)	(±1.76)	(±1.73)	(±1.57)	(±1.30)	(±1.15)	(±1.58)	(±0.98)
TD	1.75	1.95	2.06	1.79	1.71	1.82	1.83	1.69
	(±1.14)	(±1.38)	(±1.37)	(±1.73)	(±1.24)	(±1.69)	(±1.24)	(±1.49)

Note. ASD = Autism Spectrum Disorder; TD = Typically Developing

Supplement 4B: Multivariate tests, 700 – 1200 ms, parietal clusters, 4th image

	F (1, 38)	***p***	***η²***
Condition	1.683	0.202	0.042
Condition * IQ	1.662	0.205	0.042
Condition * Group	0.504	0.482	0.013
Ending	0.123	0.728	0.003
Ending * IQ	0.025	0.874	0.001
Ending * Group	**4,673**	**0.037***	**0.110**
Hemisphere	0.060	0.808	0.002
Hemisphere * IQ	0.131	0.720	0.003
Hemisphere * Group	1.226	0.275	0.031
Condition * Ending	3.011	0.091	0.073
Condition * Ending * IQ	3.541	0.068	0.085
Condition * Ending * Group	**4.430**	**0.042***	**0.104**
Condition * Hemisphere	2.254	0.133	0.058
Condition * Hemisphere * IQ	2.064	0.159	0.052
Condition * Hemisphere * Group	0.394	0.534	0.010
Ending * Hemisphere	0.728	0.399	0.019
Ending * Hemisphere * IQ	1.239	0.027	0.032
Ending * Hemisphere * Group	2.329	0.135	0.058
Condition * Ending * Hemisphere	0.055	0.816	0.001
Condition * Ending * Hemisphere * IQ	0.041	0.841	0.001
Condition * Ending * Hemisphere * Group	0.092	0.764	0.002
IQ	2.322	0.136	0.058
Group	**14.888**	**0.000***	**0.282**

Note. Significant effects are printed in bold and marked with an asterisk.

Supplement 4C: significant post-hoc comparisons for the Group*Ending interaction in the parietal cluster

Effect (Mean ± SD)	***p***
i_{ASD} (M = 0.64 ± 1.47 µV) < c_{TD} (M = 1.81 ± 1.36 µV)	< 0.001
i_{ASD} (M = 0.64 ± 1.47 µV) < i_{TD} (M = 1.81 ± 1.46 µV)	< 0.001
i_{ASD} (M = 0.64 ± 1.47 µV) < c_{ASD} (M = 1.81 ± 1.43 µV)	0.03

Note. ASD = Autism Spectrum Disorder; TD = Typically Developing; c= correct endings; i = incorrect endings.

Supplement 4D: significant post-hoc comparisons for the Group*Condition*Ending interaction in the parietal cluster

Effect (Mean ± SD)	***p***
IA-i_{ASD} (M = 0.38 ± 1.65 µV) < IA-c_{TD} (M = 1.85 ± 1.26 µV)	0.01
IA-i_{ASD} (M = 0.38 ± 1.65 µV) < IA-i_{TD} (M = 1.92 ± 1.55 µV)	0.01
IA-i_{ASD} (M = 0.38 ± 1.65 µV) < PC-c_{TD} (M = 1.76 ± 1.47 µV)	0.03
IA-i_{ASD} (M = 0.38 ± 1.65 µV) < PC-i_{TD} (M = 1.85 ± 1.37 µV)	0.03
IA-i_{ASD} (M = 0.38 ± 1.65 µV) < IA-c_{ASD} (M = 1.85 ± 1.64 µV)	0.01

Note. ASD = Autism Spectrum Disorder; TD = Typically Developing; c= correct endings; i = incorrect endings.

Supplement 5A: Mean (±SD) area (µV*ms), 700 – 1200 ms, parietal clusters, 4th image

	Intention Attribution		***Physical Causality***	
Ending	***Congruous***	***Incongruous***	***Congruous***	***Incongruous***
ASD	0.67 (±2.67)	2.53 (±3.52)	0.81 (±1.92)	1.26 (±2.07)
TD	-0.53 (±1.41)	0.74 (±1.34)	-0.93 (±1.78)	0.51 (±2.02)

Note. ASD = Autism Spectrum Disorder; TD = Typically Developing.

Supplement 5B: Multivariate tests, 700 – 1200 ms, parietal clusters, 4th image

	F (1, 38)	*p*	*η²*
Condition	0.144	0.706	0.004
*Condition * IQ*	0.049	0.826	0.001
*Condition * Group*	0.176	0.678	0.005
Ending	0.812	0.373	0.021
*Ending * IQ*	1.720	0.198	0.043
*Ending * Group*	0.001	0.976	0.000
*Condition * Ending*	3.002	0.091	0.073
*Condition * Ending * IQ*	3.568	0.067	0.086
Condition * Ending * Group	**5.326**	**0.027***	**0.123**
IQ	0.002	0.961	0.000
Group	**6.763**	**0.013***	**0.151**

Note. Significant effects are printed in bold and marked with an asterisk.

Supplement 5C: significant post-hoc comparisons for the Group*Condition*Ending interaction in the frontal cluster

Effects (Mean ± SD)	*p*
IA-i_{ASD} (M = 2.53 ± 3.52 µV) > IA-c_{TD} (M = -0.53 ± 1.41 µV)	0.01
IA-i_{ASD} (M = 2.53 ± 3.52 µV) > PC-c_{TD} (M = -0.93 ± 1.78 µV)	0.00
IA-i_{ASD} (M = 2.53 ± 3.52 µV) > IA-c_{ASD} (M = 0.67 ± 2.67 µV)	0.00
IA-i_{ASD} (M = 2.53 ± 3.52 µV) > PC-c_{ASD} (M = 0.81 ± 1.92 µV)	0.01
IA-i_{TD} (M = 0.74 ± 1.34 µV) > PC-c_{TD} (M = -0.93 ± 1.78 µV)	0.01
PC-i_{TD} (M = 0.51 ± 2.02 µV) > PC-c_{TD} (M = -0.93 ± 1.78 µV)	0.03

Note. ASD = Autism Spectrum Disorder; TD = Typically Developing; c= correct endings; i = incorrect endings.
